# Protection Against Epithelial Damage During *Candida albicans* Infection Is Mediated by PI3K/Akt and Mammalian Target of Rapamycin Signaling

**DOI:** 10.1093/infdis/jit824

**Published:** 2013-12-19

**Authors:** David L. Moyes, Chengguo Shen, Celia Murciano, Manohursingh Runglall, Jonathan P. Richardson, Matthew Arno, Estibaliz Aldecoa-Otalora, Julian R. Naglik

**Affiliations:** 1 Department of Oral Immunology, King's College London Dental Institute; 2 Genomics Centre, King's College London, United Kingdom

**Keywords:** Akt, *Candida albicans*, epithelial, inflammation, fungal, PI3 kinase, damage, MAPK, c-Fos, microarray, mTOR

## Abstract

***Background.*** The ability of epithelial cells (ECs) to discriminate between commensal and pathogenic microbes is essential for healthy living. Key to these interactions are mucosal epithelial responses to pathogen-induced damage.

***Methods.*** Using reconstituted oral epithelium, we assessed epithelial gene transcriptional responses to *Candida albicans* infection by microarray. Signal pathway activation was monitored by Western blotting and transcription factor enzyme-linked immunosorbent assay, and the role of these pathways in *C. albicans*–induced damage protection was determined using chemical inhibitors.

***Results.*** Transcript profiling demonstrated early upregulation of epithelial genes involved in immune responses. Many of these genes constituted components of signaling pathways, but only NF-κB, MAPK, and PI3K/Akt pathways were functionally activated. We demonstrate that PI3K/Akt signaling is independent of NF-κB and MAPK signaling and plays a key role in epithelial immune activation and damage protection via mammalian target of rapamycin (mTOR) activation.

***Conclusions.*** PI3K/Akt/mTOR signaling may play a critical role in protecting epithelial cells from damage during mucosal fungal infections independent of NF-κB or MAPK signaling.

The ability of mucosal surfaces to discriminate commensal from pathogenic organisms is critical in maintaining health. A distinguishing feature of mucosal pathogens over commensals is their ability to cause epithelial damage [1] and apart from activating immune responses, an essential function of epithelial cells (ECs) is damage limitation.

Innate immune response activation is mediated by pattern recognition receptors (PRRs) via intracellular signal pathways, including nuclear factor kappa B (NF-κB), phosphoinositide 3 kinase (PI3K), mitogen-activated protein kinase (MAPK) pathways [2, 3], nuclear factor of activated T cells (NFAT), and interferon regulatory factors (IRFs) [3, 4]. Notably, in myeloid cells, PI3K signaling is associated with suppression of PRR-mediated responses [2, 5]. However, the role of the PI3K pathway in epithelial PRR responses is unclear. Likewise, the epithelial intracellular signaling pathways associated with protecting ECs against microbial-induced damage are poorly understood. Studies using bacteria show protective responses mediated via p38 signaling [6], and NF-κB and MAPK signaling are associated with inflammation-induced cell death [7, 8]. However, host signaling pathways associated with protecting ECs against fungal-induced damage remain unidentified.

The opportunistic human fungal pathogen *Candida albicans* is a normal microbiota constituent in approximately 50% of individuals but causes mucosal diseases with significant morbidity in immunocompromised hosts [9]. Most host–fungal interaction studies have been performed using myeloid cells, but ECs can also play an active role [10–14]. Understanding how ECs interact with *C. albicans* is of paramount importance in determining how ECs respond to fungal-induced damage. Earlier work characterized the global transcriptional changes in *C. albicans* cells during infection of oral ECs [15]; however, the transcriptional profile of ECs during *C. albicans* infection has only recently been addressed [16].

Using targeted proteomics, we previously identified that *Candida* species activate MAPK and NF-κB signaling in oral and vaginal ECs [11, 17–19]. We discovered that yeast/hyphal discrimination was dependent on MAPK-p38 and constituted activation of proinflammatory cytokines via the c-Fos transcription factor [11, 17]. Furthermore, this EC activation mechanism results in neutrophil-dependent, epithelial Toll-like receptor (TLR4)–mediated protection of mucosal surfaces against *C. albicans* infection [10].

In this study, we used gene expression profiling to determine which signaling pathways are activated by *C. albicans* infection of oral epithelium. We discovered that in addition to MAPK and NF-κB, PI3K/Akt signaling is also activated by *C. albicans*. Although PI3K/Akt signaling is not associated with EC discrimination of *C. albicans* morphology, it is involved in regulation of granulocyte macrophage colony-stimulating factor (GM-CSF) and granulocyte colony-stimulating factor (G-CSF) secretion independent of MAPK or NF-κB signaling. Crucially, PI3K/Akt signaling activates damage protection cellular processes in response to *C. albicans*.

## MATERIALS AND METHODS

### Cell Lines and Reagents

All monolayer experiments were performed using the TR146 buccal epithelial carcinoma cell line. Cells were cultured in Dulbecco's modified Eagle medium 10%/fetal calf serum 1% penicillin/streptomycin. Cells were serum starved overnight and infections performed in serum-free conditions. Reconstituted human oral epithelium (ROE) created using TR146 cells was purchased from SkinEthic Laboratories and used as previously described [10]. Wortmannin, LY294002, Ku-63794, and SB203580 were from Calbiochem. All inhibitors were dissolved in dimethyl sulfoxide (DMSO) with equivalent quantities of DMSO used as vehicle controls. Antibodies to phospho-c-Jun, phospho-MKP1 phospho-Akt, phospho-PDK1, phospho-IκBα, phospho-GSK3β, phospho-IRF3, phospho-STAT3, and c-Fos were purchased from Cell Signaling Technologies (New England Biolabs). Antibody to α-actin was purchased from Millipore. The *C. albicans* SC5314 strain was used in all experiments [11, 17].

### RNA Isolation and Analysis

RNA was isolated using the GenElute total mammalian RNA miniprep kit (Sigma). Genomic DNA contamination was removed using the Turbo DNase free kit (Ambion). For microarray analysis, RNA was amplified using the MessageAmp Premier RNA Amplification Kit (Ambion), then hybridized onto U133a 2.0 gene chips (Affymetrix) after fragmentation. Chips were scanned (Affymetrix GeneChip Scanner 3000) and checked using Affymetrix Command Console (AGCC) software suite. These data was statistically analyzed using Partek Genomics Suite (version 6.4). Gene Ontology analysis was performed using MetaCore (version 2.4, GeneGo Inc). Real-time reverse transcription polymerase chain reaction (qRT-PCR) analysis was carried out using primers listed in Table 1, primers and probes previously described for TLR5 [20], or assay-on-demand set for YWHAZ (Applied Biosystems). Reactions were performed using a Rotogene 6000 (Qiagen) for 45 cycles of 95°C for 5 seconds and 60°C for 20 seconds. Data were analyzed using the 2 standard curve method.

**Table 1.** Primers Used for Quantitative Polymerase Chain Reaction Validation

Gene	Sense	Antisense
*mmp1*	5′–ACTCTGGAGTAATGTCACACCT–3′	5′–GTTGGTCCACCTTTCATCTTCA–3′
*mmp10*	5′–CCCACTCTACAACTCATTCACAG–3′	5′–TCAGATCCCGAAGGAACAGAT–3′
*timp-1*	5′ - GGGTTCCAAGCCTTAGGGG–3′	5′–TTCCAGCAATGAGAAACTCCTC3′ -
*cox2*	5′ - ATATGTTCTCCTGCCTACTGGAA–3′	5′–GCCCTTCACGTTATTGCAGATG–3′
*dusp1*	5′ - GGCCCCGAGAACAGACAAA–3′	5′–GTGCCCACTTCCATGACCAT–3′
*dusp6*	5′ - ACACCCCTCCTTGCTGGAAT–3′	5′–CACACACAAAGAAAGCAGCCC–3′
*dusp5*	5′ - GCGACCCACCTACACTACAAA–3′	5′–CTTCATAAGGTAAGCCATGCAGA–3′
β-*def4*	5′ - GGTGGTATAGGCGATCCTGTT–3′	5′–AGGGCAAAAGACTGGATGACA–3′
*c-fos*	5′–GGGCAAGGTGGAACAGTTATC–3′	5′–CCGCTTGGAGTGTATCAGTCA–3′

### Immunoblotting

Cells were lysed as previously described [11] using RIPA lysis buffer containing protease (Sigma) and phosphatase inhibitor cocktails (Perbio). Protein content was assayed using a bicinchoninic acid protein assay (Perbio) and 15 µg was separated on 12% sodium dodecyl sulfate polyacrylamide gel electrophoresis gels, transferred to polyvinylidene fluoride (GE Healthcare), probed with primary and secondary antibodies, and then developed using an Enhanced Chemiluminescent substrate (Millipore) before being exposed to photographic film (GRI Ltd).

### Transcription Factor Analysis

Nuclear proteins were isolated from cells using a nuclear protein extraction kit (Active Motif). Protein levels were assayed as above and 5 µg was used in a TransAM enzyme-linked immunosorbent assay (Active Motif, Belgium).

### Cytokine Determination

Cytokine levels were determined using the Fluorokine microbead assay system (R&D Systems) and measured on a Bioplex-200 machine (Bio-Rad).

### Epithelial Cell Damage Determination

EC damage was determined by measuring lactate dehydrogenase (LDH) activity in cell-culture supernatant using the Cytotox 96 nonradioactive cytotoxicity assay (Promega).

### Statistical Analysis

Data were analyzed using the 2-tailed *t* test. In all cases, *P* < .05 was taken to be significant.

## RESULTS

### Microarray Gene Expression Analysis of *C. albicans*–Infected Reconstituted Human Epithelium

The transcriptional response of *C. albicans* during EC interactions has been categorized into 3 phases: early attachment phase (1–3 hours), intermediate invasion phase (3–12 hours), and late infection phase (12–24 hours) [15]. Here, we determined EC transcriptional responses during the intermediate (6 hours) and late (24 hours) phases in the *C. albicans*–ROE model using microarrays. Whole-genome expression of 3 independent ROEs treated for 6 hours or 24 hours with 10^7^ colony-forming units/mL of *C. albicans* yeast cells (epithelial cells induce a rapid switch of yeast to hyphal growth by 1–2 hours [21]) or phosphate-buffered saline (PBS) was analyzed. The pattern of gene transcription revealed clear differences between the 2 different time points relative to PBS controls (Supplementary Figure 1*A*). A total of 266 genes were significantly (*P* < .001) altered at least 2-fold at 6 hours and 2887 genes at 24 hours, with 233 genes altered at both time points (Supplementary Figure 1*B*). At 6 hours postinfection, 205 genes were up-regulated and 62 genes down-regulated, whereas at 24 hours postinfection, 1126 genes were up-regulated and 1782 genes down-regulated (Supplementary Figure 1*B*). At both time points, 183 genes were up-regulated and 44 genes down-regulated (Supplementary Figure 1*B*). Multiple cytokine, signaling, and inflammatory genes were up-regulated at both time points (Table 2). Analysis of cell damage showed significant increases in LDH levels only after 12 hours (Supplementary Figure 2), indicating that the majority of damage occurs during the late phase of infection.

**Table 2.** Selected Genes Showing Significant Up-regulation in Reconstituted Human Oral Epithelium 6 Hours and 24 Hours After Infection With *Candida albicans*

Gene	6 Hours Postinfection		24 Hours Postinfection	
	*P* Value	Fold Change	*P* Value	Fold Change
Transcription factors				
FOS (c-Fos)	5.61 × 10^−03^	3.5		nc
EGR3	7.03 × 10^−04^	4.3	2.75 × 10^−05^	32.4
FOSL1 (Fra1)	7.88 × 10^−05^	7	1.05 × 10^−05^	12.9
ATF3	5.81 × 10^−04^	4.2	5.56 × 10^−05^	8.4
ELK3		nc	1.47 × 10^−05^	5.4
EGR1	4.61 × 10^−06^	4	3.38 × 10^−04^	5.3
JUN		nc	4.54 × 10^−05^	4.0
FOSB		nc	5.35 × 10^−04^	3.2
CREB1		nc	8.26 × 10^−04^	2.7
MAPK modulators				
DUSP1 (MKP1)	1.95 × 10^−04^	4.7	2.27 × 10^−05^	7.6
DUSP5 (HVH3)	2.6 × 10^−04^	5.6	1.19 × 10^−04^	7.0
DUSP6 (MKP3)	9.14 × 10^−05^	8.8	8.86 × 10^−04^	6.8
SPRY2	2.93 × 10^−04^	4.4	1.33 × 10^−04^	5.9
TRIB1	1.9 × 10^−04^	4.8	5.38 × 10^−05^	4.7
SPRY4	4.72 × 10^−03^	3	9.49 × 10^−05^	4.7
Signaling molecules				
INPP4B			1.42 × 10^−05^	10.9
ITPKC	6.15 × 10^−04^	4		nc
GADD45A		nc	9.62 × 10^−05^	5.4
SOCS1	7.91 × 10^−04^	2.6	7.11 × 10^−05^	5.0
TNFAIP3	5.79 × 10^−03^	3.2	1.03 × 10^−04^	4.7
PITPNC1		nc	4.28 × 10^−04^	4.0
NFKBIA		nc	8.71 × 10^−06^	3.0
NFKBIE		nc	5.66 × 10^−05^	3.0
NFAT5		nc	3.6 × 10^−04^	3.0
SOCS3		nc	6.98 × 10^−05^	2.8
BCL10		nc	1.9 × 10^−04^	2.5
TRIF		nc	4.49 × 10^−05^	2.4
IL-1 family cytokines				
IL-1F9	3.8 × 10^−04^	8.1	9.59 × 10^−05^	34.7
IL-1α	2.16 × 10^−03^	6.6	1.11 × 10^−04^	10.0
IL-1β	1.63 × 10^−03^	3.6	1.16 × 10^−04^	7.9
IL-1F5		nc	1.53 × 10^−04^	7.0
IL-1ra		nc	8.51 × 10^−04^	6.1
IL-6 family cytokines				
CLCF-1	1.32 × 10^−05^	6	2.09 × 10^−05^	12.7
LIF	2.66 × 10^−04^	3.9	6.33 × 10^−05^	7.1
IL-6	1.53 × 10^−03^	3	5.07 × 10^−05^	5.6
Other cytokines/chemokines				
IL-8	5.12 × 10^−05^	6.5	3.45 × 10^−04^	24.2
GM-CSF			1.67 × 10^−06^	24.2
HBEGF	1.32 × 10^−05^	6.4	8.11 × 10^−06^	11.3
CCL20	8.13 × 10^−04^	6.8	5.40 × 10^−08^	10.6
IL-11	6.48 × 10^−03^	2.3	2.5 × 10^−04^	6.3
IL-24		nc	2.80 × 10^−05^	4.3
Surface receptors				
CEACAM6		nc	2.60 × 10^−06^	42.1
CEACAM1		nc	1.07 × 10^−05^	15.6
PTX3	5.83 × 10^−03^	5.3	5.15 × 10^−05^	11.2
ICAM1	6.65 × 10^−03^	3.5	1.05 × 10^−05^	5.6
TLR5		nc	2.4 × 10^−03^	−8.1
CLEC7A		nc	3.13 × 10^−04^	−9.2
Proteases/inhibitors				
SERPINB2	8.20 × 10^−05^	7.6	1.06 × 10^−04^	42.3
MMP10	4.27 × 10^−04^	3.5	4.30 × 10^−06^	28.1
MMP1	1.02 × 10^−03^	5.7	1.79 × 10^−06^	17.7
SERPINB1		nc	6.1 × 10^−04^	7.9
TIMP1		nc	3.54 × 10^−05^	3.9
Apoptosis				
BCL2A1	8.51 × 10^−03^	3.3	6.4 × 10^−04^	20.0
BCL2L1		nc	4.4 × 10^−04^	2.2
CASP8		nc	6.89 × 10^−04^	−2.1
BCLAF1		nc	1.51 × 10^−04^	−3.7
Antimicrobial peptides				
DEFB4		nc	1.69 × 10^−04^	30.8
S100P		nc	1.36 × 10^−06^	6.4
S100A12		nc	9.85 × 10^−04^	3.2
Interferon-stimulated genes				
ISG20		nc	6.54 × 10^−05^	4.9
PRKRIR		nc	6.87 × 10^−04^	−2.1
IFI16		nc	1.34 × 10^−05^	−2.2
IFIT3		nc	2.25 × 10^−03^	−2.4
IFIT5		nc	2.87 × 10^−05^	−5.2
IFIT1		nc	3.43 × 10^−04^	−3.15

Abbreviations: GM-CSF, granulocyte macrophage colony-stimulating factor; IL, interleukin; nc, no change.

Intermediate gene up-regulation is associated with detection of fungal infection, whereas the later time point contains genes involved in prolonged EC responses to infection. At both time points, MAPK-induced transcription factors (TFs) and phosphatases, NF-κB signaling, other signaling pathway TFs, and cytokines demonstrated changes (Table 2). Up- or down-regulation of selected genes previously implicated as important/involved in inflammatory processes or EC responses to *C. albicans* was confirmed by qRT-PCR (Supplementary Figure 3).

Among the most highly up-regulated genes at both time points were MAPK phosphatases *dusp1* (MKP1)*, dusp5* (HVH3)*,* and *dusp6* (MKP3), with increased expression of other genes involved in MAPK regulation (*sprouty-2* and *-4* and *tribbles-1*)*,* as well as MAPK TFs (Fos and Jun family members) (Table 2). Other signaling and TF genes showed increased expression at 6 hours, including *egr1*, *egr3*, and *socs1. tlr5* and *dectin-1* (*clec7a*) were down-regulated whereas *ptx3* and *galectin-3* were up-regulated and TLR-associated adapters and regulators (*trif, a20*, and *itch*) showed up-regulation. We observed increased expression of cytokine genes in common with our previous studies demonstrating increased cytokine protein expression [11].

### Gene Ontology Analysis of *C. albicans*–Infected Reconstituted Human Epithelium

**Figure 1.**
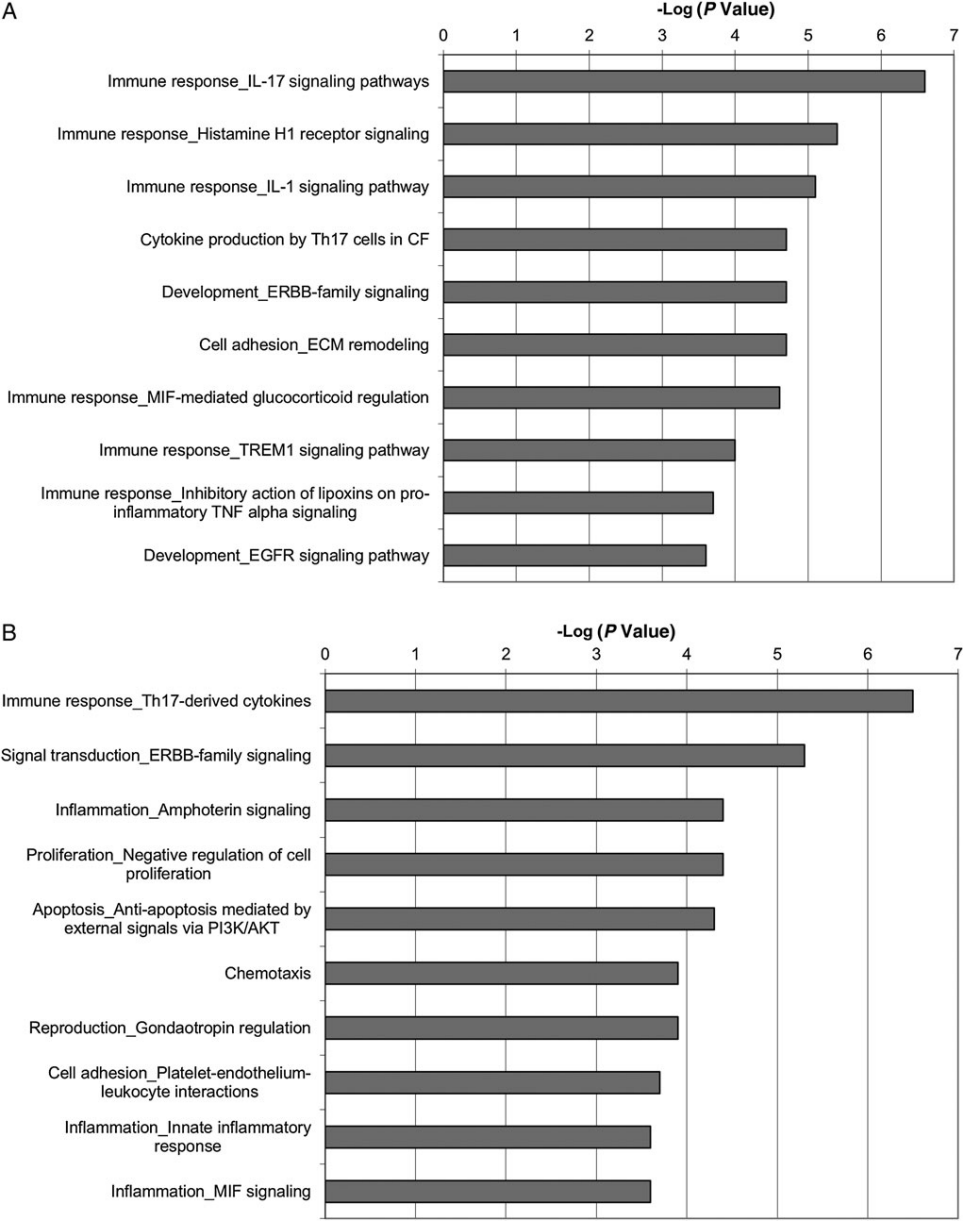
Pathways (*A*) and networks (*B*) enriched in reconstituted human oral epithelium 6 hours after infection with *Candida albicans*.

At 6 hours, several pathways showed significant enrichment in gene expression (Figure 1*A*), including immune response pathways (interleukin [IL] 17 signaling [−log *P* value = 6.6], IL-1 signaling [5.1], Macrophage migration Inhibitory Factor (MIF)-mediated glucocorticoid regulation [4.61], and Triggering Receptor Expressed on Myeloid cells-1 (TREM-1) signaling [4]). There is also significant enrichment in other pathways (ErbB family signaling [4.7], epidermal growth factor receptor [EGFR] signaling [3.6], and extracellular matrix remodeling [4.7]). Analysis of MetaCore Process Networks enrichment (Figure 1*B*) provides further evidence of immune activation, with Th17-derived cytokines (6.5), ERBB-family signaling (5.3), MIF signaling (3.6), and innate inflammatory response (3.6) among the 10 most enriched processes. Several cell survival processes show enrichment (negative regulation of cell proliferation [4.4], antiapoptosis mediated by external signals via PI3K/Akt [4.3]). These data suggest that ECs mount an immediate and robust response to *C. albicans* infection.

**Figure 2.**
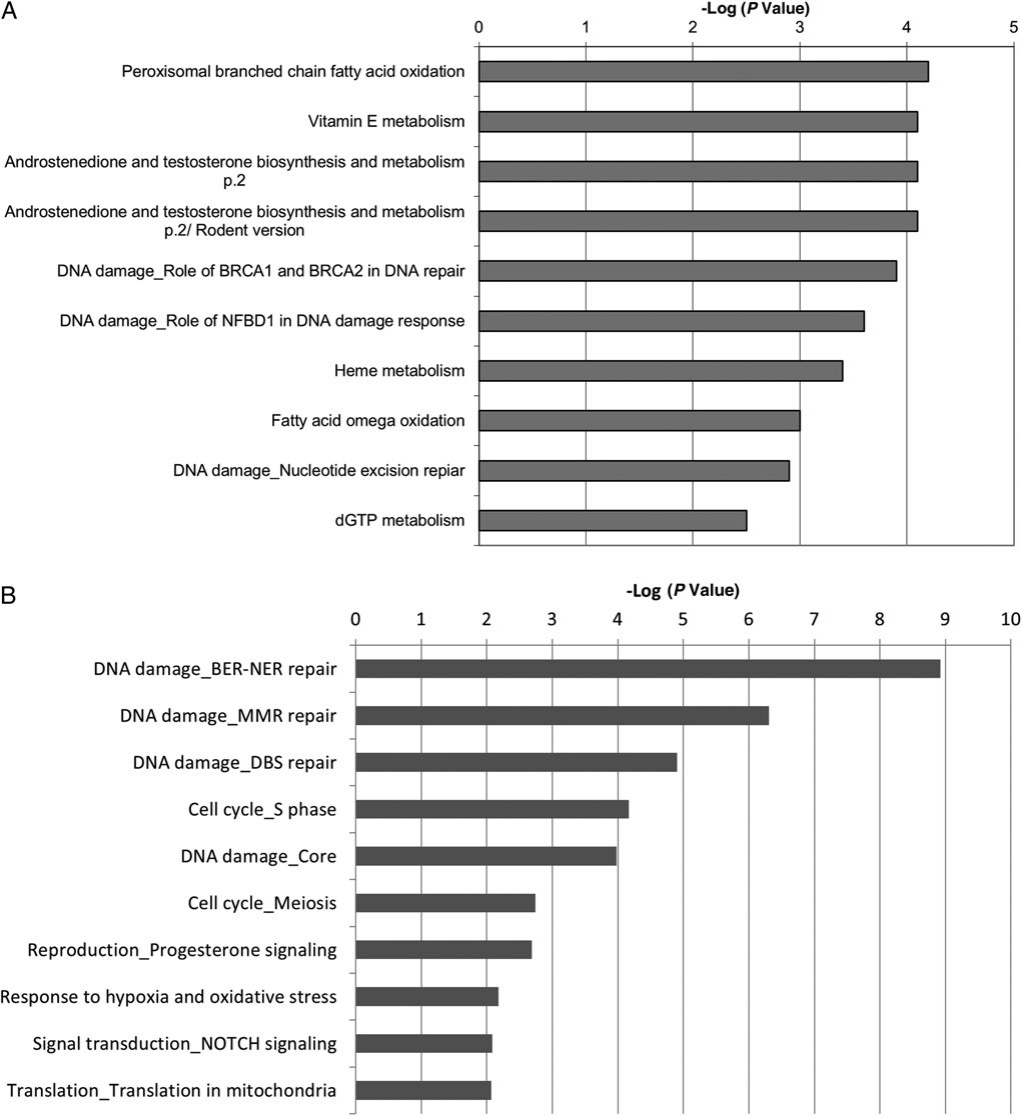
Pathways (*A*) and networks (*B*) enriched in reconstituted human oral epithelium 24 hours after infection with *Candida albicans*.

The responses 24 hours postinfection are more heterogeneous, reflecting the variety of stimuli affecting ECs at this time point. The most enriched MetaCore pathways include those involved in metabolism (Figure 2*A*), and analysis of enriched MetaCore Process Networks identifies several DNA damage-associated processes (Base Excision Repair-Nucleotide Excision Repair (BER-NER) repair [8.9], DNA Mismatch Repair (MMR) repair [6.3], DBS repair [4.9], and core processes [3.9]) (Figure 2*B*). Furthermore, there is enrichment of networks involved in cell cycle (DNA production (S phase [4.2]), likely linked with DNA repair processes. Along with enrichment in mitochondrial translation genes (translation in mitochondria [2.1]), these may represent a continuation of earlier apoptotic processes. Other enriched processes suggest changes in intercellular communication (NOTCH signaling [2.1]) and stress responses (response to hypoxia and oxidative stress [2.2]). Together, these data suggest a general response to an invasive, damage-inducing pathogen.

### *Candida albicans* Does Not Induce IRF-3, NFAT, or STAT1 Signaling in Epithelial Cells

**Figure 3.**
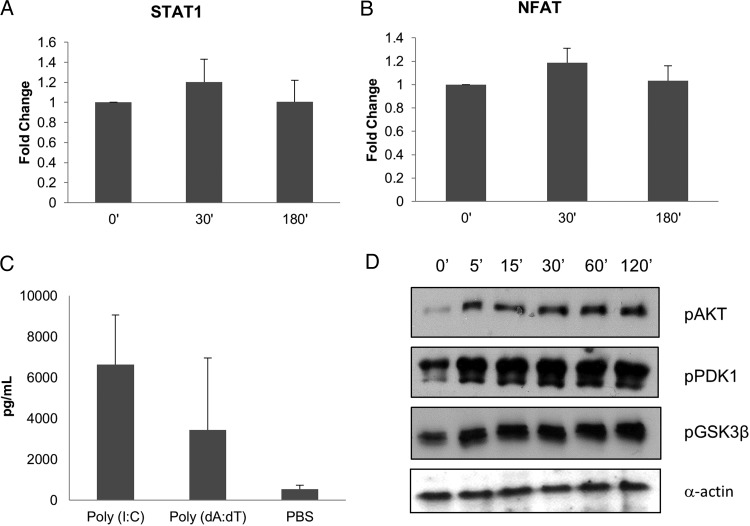
Levels of DNA-binding activity of STAT1 (*A*) and NFAT (*B*) in TR146 cells 30 minutes and 3 hours postinfection with *Candida albicans. C*, Production of granulocyte colony-stimulating factor by TR146 cells in response to TLR3 (poly I:C, 25 μg/mL) and RIG-I (Poly dA:dT, 1 μg/mL) agonists. *D*, Increasing phosphorylation of PDK1, Akt, and GSK3β from 5 minutes up to 2 hours postinfection. In all cases, multiplicity of infection = 10. *Candida albicans* was added as 100% yeast that switched to hyphal growth by 2 hours after infection. Results shown are the mean (*A*–*C*) or representative (*D*) of 3 independent experiments. Abbreviation: PBS, phosphate-buffered saline.

As well as NF-κB and MAPK signaling [11], the microarray data indicated increased IRF3 and Janus kinase/signal transducer and activator of transcription (JAK/STAT)–regulated genes, as well as increases in NFAT expression (Table 2). Therefore, we determined whether these pathways and their downstream TFs were functionally activated (phosphorylated) in response to *C. albicans* at early time points (optimum 2 hours [11]) in monolayer TR146 ECs, which comprise the ROE model. TR146 monolayers were used to maximize the signal, and times up to 2 hours were optimum to identify signal pathways driving 6-hour gene expression. Despite the well-documented link between MAPK, NF-κB, and IRF3 signaling after PRR stimulation [3], IRF3 phosphorylation was not detected up to 2 hours postinfection (data not shown), even though NF-κB and MAPK signaling is activated [11]. To confirm that this lack of response was not a result of a defect in the TLR3 or RIG-I pathways, we stimulated TR146 monolayers with the TLR3 (Poly I:C) and IPS (Poly dA:dT) agonists. Both these agonists increased the production of G-CSF from TR146 cells (Figure 3*C*), indicating that these pathways are functional in TR146 cells. STAT3 phosphorylation was not detected up to 2 hours postinfection (data not shown), and STAT1 DNA binding and transcriptional activity showed no increase at 30 minutes or 3 hours postinfection (Figure 3*A*). Finally, although NFAT signaling is associated with myeloid cell responses to *C. albicans* [4], we found no NFAT DNA-binding activity at 30 minutes or 3 hours postinfection (Figure 3*B*). Together, these data suggest that IRF3, JAK/STAT, and NFAT signaling play no role in EC responses to *C. albicans* infection.

### *Candida albicans* Infection Induces PI3K/Akt Signaling

Microarray analysis demonstrated several PI3K-activating receptor-ligand interactions (ERBB family signaling, EGFR signaling, antiapoptosis (Table 2, Figures 1 and 2). To investigate the functional significance of these observations, we looked for phosphorylation of PDK1, Akt, and GSK-3β. Immunoblot analysis of *C. albicans*–infected TR146 oral ECs demonstrated increased PDK1 phosphorylation as early as 5 minutes postinfection, peaking at 2 hours (Figure 3*D*) with a matching increase in Akt phosphorylation (Figure 3*D*). We also observed increases in GSK-3β phosphorylation with similar kinetics (Figure 3*D*).

**Figure 4.**
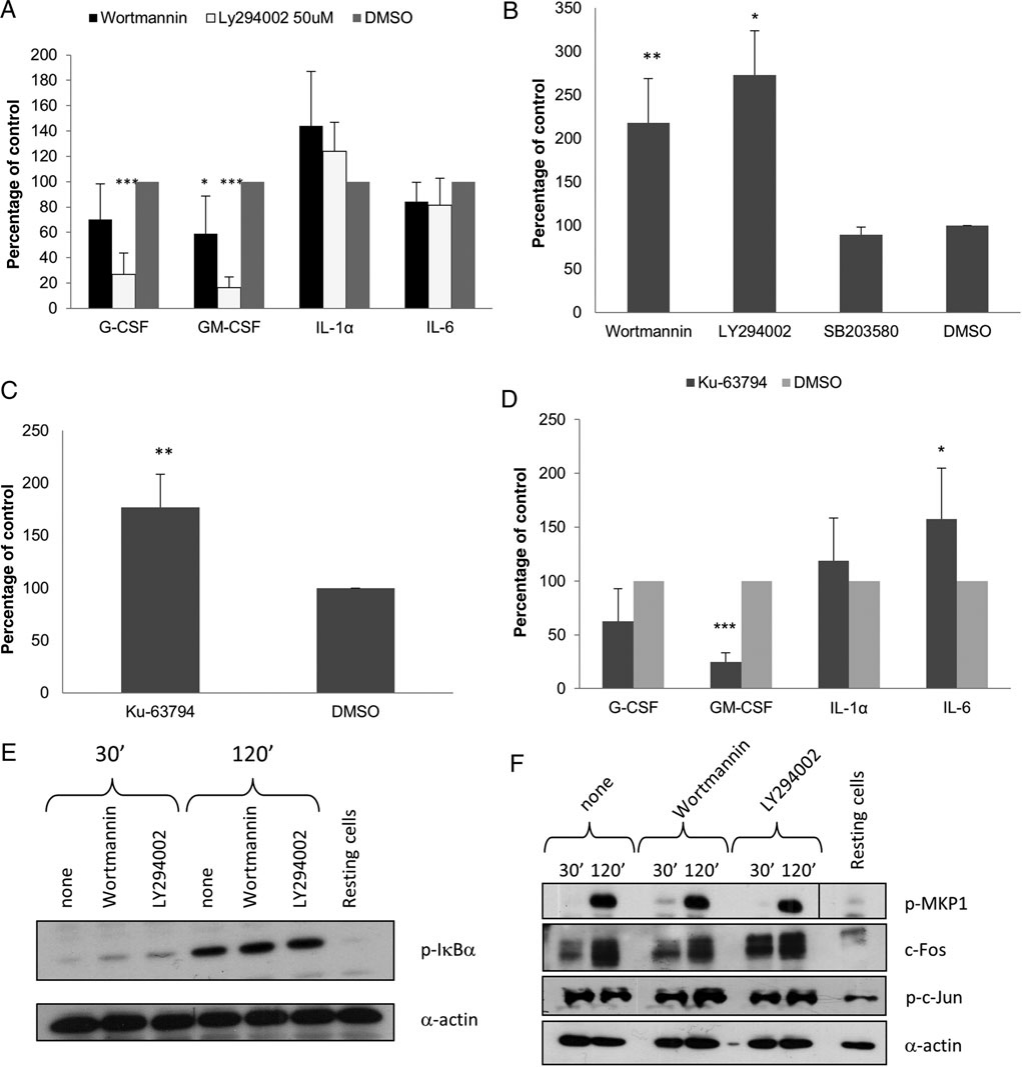
Inhibition of PI3K/Akt/mammalian target of rapamycin (mTOR) signaling induction in TR146 cells infected with *Candida albicans. A*, Effect of inhibition of PI3K/Akt signaling by 1 µM wortmannin or 50 µM LY294002 on cytokine production after 24 hours (multiplicity of infection [MOI] = 0.01) shown as percentage of the dimethyl sulfoxide (DMSO) vehicle control. *B*, Effect of inhibition of PI3K/Akt (wortmannin [1 µM] and LY294002 [50 µM]) or p38 (SB203580 [10 µM]) signaling on cell damage (lactate dehydrogenase [LDH] release) after 24 hours (MOI = 0.01) shown as percentage of the vehicle control (DMSO). *C*, Effect of inhibition of mTOR activity with 10 μM Ku-63794 on cell damage (LDH release) after 24 hours (MOI] = 0.01) shown as percentage of DMSO vehicle control. *D*, Effect of inhibition of mTOR activity with 10 μM Ku-63794 on cytokine production after 24 hours (MOI = 0.01) shown as percentage of the vehicle control (DMSO). *E*, Effect of inhibition of PI3K/Akt signaling by 1 µM wortmannin or 50 µM LY294002 on phosphorylation of IκBα 2 hours postinfection (MOI = 10). *F*, Effect of inhibition of PI3K/Akt signaling by 1 µM wortmannin or 50 µM LY294002 on phosphorylation of MKP1 and c-Jun and on the production of c-Fos 2 hours postinfection (MOI = 10). *Candida albicans* was added as 100% yeast, which switched to hyphal growth by 2 hours postinfection. Data are the mean (*A*–*D*) or representative (*E* and *F*) of at least 3 independent experiments. *P < .05, **P < .01, ***P < .001. Abbreviations: G-CSF, granulocyte colony-stimulating factor; GM-CSF, granulocyte macrophage colony-stimulating factor; IL, interleukin.

Because PI3K/Akt signaling plays a role in modulating TLR-induced cytokine production in ECs [22], we inhibited this pathway with 2 separate PI3K inhibitors, wortmannin (1 µM) and LY294002 (50 µM), to determine its functional significance in EC cytokine responses. Both inhibitors significantly reduced GM-CSF levels (*P* < .05 and *P* < .001; Figure 4*A*), whereas LY294002 additionally reduced G-CSF levels (*P* < .001). In contrast, IL-1α production was increased, although not significantly (Figure 4*A*), whereas IL-6 production was unaffected. Given that IL-1α production is associated with damage [23], these data suggest a role for PI3K/Akt signaling in protection/prevention of damage by *C. albicans*. Therefore, we analyzed 24 hours postinfection supernatant LDH levels with or without inhibition, finding significantly increased LDH release (Figure 4*B*). Inhibition of p38 signaling had no effect on LDH release (Figure 4*B*), indicating that despite mediating discrimination between *C. albicans* yeast and hyphae via c-Fos [11], p38 plays no role in EC damage protection in fungal infection.

The Akt target, mammalian target of rapamycin (mTOR), plays an important role in cell survival [24]. Therefore, to determine whether the protective effect regulated by PI3K/Akt signaling was mediated via mTOR, we inhibited mTOR and found a significant increase in LDH release (*P* < .01), indicating increased cell damage (Figure 4*C*). As with PI3K/Akt inhibition, levels of G-CSF (*P* < .05) and GM-CSF (*P* < .001) were significantly reduced (Figure 4*D*). However, unlike Akt inhibition, there was a significant increase in IL-6 production (*P* < .05). The increased damage in PI3K/Akt/mTOR-inhibited cells was not due to toxic effects of the inhibitors, as treatment with inhibitors alone did not result in a significant increase in LDH release (data not shown).

### MAPK and NF-κB Signaling Induced by *C. albicans* Is Independent of the PI3K Pathway

PI3K/Akt signaling can modulate activation of NF-κB and AP-1 TFs [2, 25]. Thus, we investigated whether activation of PI3K/Akt signaling modulates NF-κB (IκBa) or MAPK (c-Jun, MKP1, c-Fos) responses to *C. albicans* infection as previously identified [11]. PI3K/Akt inhibition had no effect on IκBα, phospho-c-Jun, or MKP1 phosphorylation or c-Fos production at 30 minutes or 2 hours (Figure 4*E* and 4*F*), indicating that NF-κB and MAPK signaling is independent of PI3K/Akt signaling in response to fungal infection.

## DISCUSSION

In this study, we describe the global transcriptional response of oral ECs to *C. albicans* infection and identify PI3K/Akt signaling in addition to MAPK and NF-κB signaling as the major epithelial response pathways against this fungus. PI3K/Akt signaling is independent of NF-κB and MAPK signaling, and although it plays a minor role in inducing EC proinflammatory responses, PI3K/Akt signaling may play a key role in protecting ECs from *C. albicans*–induced damage.

The transcriptome of *C. albicans*–infected ROE reveals interesting findings. At 6 hours postinfection, several enriched immune response pathways were related to cytokine and other receptor signaling pathways but, notably, neither TLR or other conventional fungal PRR receptor pathways were enriched, supporting our previous findings implying no role for TLRs or other conventional PRRs in early recognition of *C. albicans* by ECs [11]. In contrast, ERBB family signaling showed significant enrichment, and previous studies support a role for EGFR-ERBB1 signaling during *C. albicans* infections [26, 27]. Although the TREM-1 signaling pathway was enriched, we found no increase in the PRR *TREM-1* expression. Thus, this enrichment possibly represents an increase in PRR signaling-associated gene expression. Enrichment of amphoterin and IL-1 signaling pathways suggests involvement of damage-associated molecular patterns in EC responses to *C. albicans*. This is expected given the stress/damage resulting from tissue invasion. Together, these pathways probably form the core EC response to fungal invasion and damage. The increase in the protease inhibitor SerpinB2 is interesting but should be interpreted with caution as a role has yet to be identified in host infection responses [13].

The 24-hour pathways and networks show enrichment in cell survival, DNA repair, or metabolism, reflecting differing EC priorities during late infection, predominantly targeting apoptosis and cell growth. Along with increases in tissue remodeling genes, this suggests a general damage repair mechanism, maintaining mucosal barrier integrity. The late appearance of apoptosis genes suggests that apoptotic mechanisms are induced at later stages of infection, as previously reported for *C. albicans* [28, 29] and bacterial EC infection [30].

In the intermediate infection stage (6 hours), NF-κB–induced genes were up-regulated, which was expected given this pathway's role in EC antifungal responses [11]. Although no MAPK proteins showed increased expression, we observed increases in expression of MKP1 (*dusp1*) and other members of this family—HVH3 (*dusp5*) and MKP3 (*dusp6*). As with MKP1, these proteins negatively regulate MAPK signaling, but with different specificity, acting to dephosphorylate ERK1/2 [31]. Given their high degree of up-regulation, these phosphatases potentially form the core MAPK-regulatory response mechanism during *C. albicans* infection. Other up-regulated MAPK regulators include 2 Sprouty genes, *spry2* and *spry4*, involved in regulating ERK1/2 signaling [7, 32], and *trib1* controlling activation of MAPKs by MAP2Ks [33] and involved in antifungal responses of *Caenorhabditis elegans* [34].

In addition to MAPK regulatory factors, we observed up-regulation of several AP-1 proteins, persisting throughout infection, confirming a central role for AP-1 in EC antifungal responses [11]. In particular, ATF3, a transcriptional repressor modulating responses to infection [35], is upregulated early, possibly as part of a negative feedback loop. Alternatively, ATF3 may work in conjunction with other TFs, allowing for shifts in gene expression profile during infection.

Along with MAPK and NF-κB pathways, other signaling pathways and TFs show upregulation after *C. albicans* infection, most notably EGR1 and EGR3. These proteins are associated with myeloid cell responses to *C. albicans* [4, 36], playing a role in NFAT signaling via dectin-1, although their role in fungal EC responses is unknown. Elevation of EGR1 and EGR3 despite a lack of signaling via dectin-1, Syk [11], or NFAT (this study) implies that EGR gene activation in ECs occurs via different pathways, potentially via ERK1/2 [13, 31]. Other genes associated with PRR regulation and immune response signals are upregulated, particularly those involved in ubiquitin signaling, including A20 and ITCH [37].

The observed discrepancy between microarrays and functional studies reflects the difference between RNA expression and subsequent protein activation, with many signaling pathways showing crossover in target genes. Microarray data represent an expression snapshot, whereas signal activation data from 0–3 hours postinfection represent EC responses to *C. albicans* rather than subsequent secreted components. Despite the microarray evidence, only PI3K/Akt signaling via PDK1/Akt appears to be functionally activated alongside MAPK and NF-κB, with no evidence for activation of IRF, STATs, or NFAT. Required for dectin-1 signaling in myeloid cells [4, 36], the lack of NFAT signaling confirms our previous findings that dectin-1 is not involved in EC recognition of *C. albicans* [11]. Induction of PI3K/Akt pathway activity in ECs was expected, given the wide range of stimuli and receptors reported to activate this pathway [38] and the demonstrated role of PI3K/Akt signaling in monocytes during fungal immunity [39, 40], TLR-mediated pathogen detection [13], and cytokine secretion [22, 41]. Here, we demonstrate PI3K/Akt/mTOR signaling is important in regulating G-CSF and GM-CSF production from ECs. Notably, these cytokines are associated with mucosal healing, having been used to treat damaged mucosa [42, 43]. In contrast, IL-1α production increased when PI3K/Akt/mTOR signaling was inhibited, correlating with increased EC damage, implying that PI3K/Akt/mTOR signaling may play a role in protecting ECs from damage induction during *C. albicans* infection. Given that PI3K/Akt signaling also protects macrophages against fungal-induced killing [40], our findings indicate that PI3K/Akt signaling may represent a common mechanism by which host cells protect against pathogen-induced damage. Furthermore, as p38 inhibition did not affect damage, EC damage protection is probably independent of the p38/c-Fos hyphal recognition response we previously reported [11]. Evidence from the MetaCore ontology analysis of microarray data in this study suggests that this protection may be induced by inhibiting apoptosis in infected cells, given the enrichment in PI3K-induced antiapoptosis pathways.

PI3K/Akt signaling may be involved in other EC response mechanisms connected to damage, for example, fungal invasion. *Candida albicans*–induced endocytosis in ECs is clathrin-dependent, and PI3K/Akt signaling is thought to be crucial for this process [44] and for microbe endocytosis [45]. Interestingly, although PI3K/Akt signaling appears to initiate EC damage protection/repair mechanisms, in contrast to myeloid cell PRR ligation [25, 46, 47], it has no effect on NF-κB or MAPK signaling. Consequently, the roles of PI3K/Akt signaling probably differ between myeloid and ECs.

It is important to note that this work was carried out using the TR146 carcinoma cell line and thus may not accurately reflect findings in a normal host. However, this cell line has been used extensively in *C. albicans* infection studies of both monolayers and organotypic models [11, 18, 19, 21, 48–50] and has been found to give comparable data to those obtained from patient biopsies [11].

Our data suggest that the role of PI3K/Akt signaling in hyphal discrimination is minimal but may constitute a major damage protection response in ECs. We therefore propose a model whereby (1) p38/c-Fos signaling identifies *C. albicans* hyphae [11, 17], (2) p38/c-Fos and NF-κB drive cytokine production [11, 17], (3) ERK1/2 signaling (via MKPs) regulates p38/c-Fos activation [11, 17], and (4) PI3K/Akt/mTOR signaling induces cell protection responses (this study). Together, these EC signaling pathways act in concert to recognize potential fungal threats, initiating protective cellular responses limiting mucosal fungal infections. This study identifies PI3K/Akt/mTOR signaling, a key component of this mechanism, as potentially an important target for future antifungal therapy development.

## Supplementary Data

Supplementary materials are available at *The Journal of Infectious Diseases* online (http://jid.oxfordjournals.org/). Supplementary materials consist of data provided by the author that are published to benefit the reader. The posted materials are not copyedited. The contents of all supplementary data are the sole responsibility of the authors. Questions or messages regarding errors should be addressed to the author.

Supplementary Data
