# Supplementary material for: Protection Against Epithelial Damage During Candida albicans Infection Is Mediated by PI3K/Akt and Mammalian Target of Rapamycin Signaling
Source: J Infect Dis. 2013 Dec 19;209(11):1816–26. doi: 10.1093/infdis/jit824 (PMC4017362; doi:10.1093/infdis/jit824)
Supplement: Supplementary Data [file supp_209_11_1816__index.html]

Protection Against Epithelial Damage During Candida albicans Infection Is Mediated by PI3K/Akt and Mammalian Target of Rapamycin Signaling — Protection Against Epithelial Damage During Candida albicans Infection Is Mediated by PI3K/Akt and Mammalian Target of Rapamycin Signaling — Supplementary Data 

# Protection Against Epithelial Damage During *Candida albicans* Infection Is Mediated by PI3K/Akt and Mammalian Target of Rapamycin Signaling

## Supplementary Data

Supplementary Data

**Files in this Data Supplement:**

- Supplementary Figure 1 - tif file
- Supplementary Figure 2 - tif file
- Supplementary Figure 3 - tif file
