# Supplementary figures and images for: Protection Against Epithelial Damage During Candida albicans Infection Is Mediated by PI3K/Akt and Mammalian Target of Rapamycin Signaling
Source: J Infect Dis. 2013 Dec 19;209(11):1816–26. doi: 10.1093/infdis/jit824 (PMC4017362; doi:10.1093/infdis/jit824)

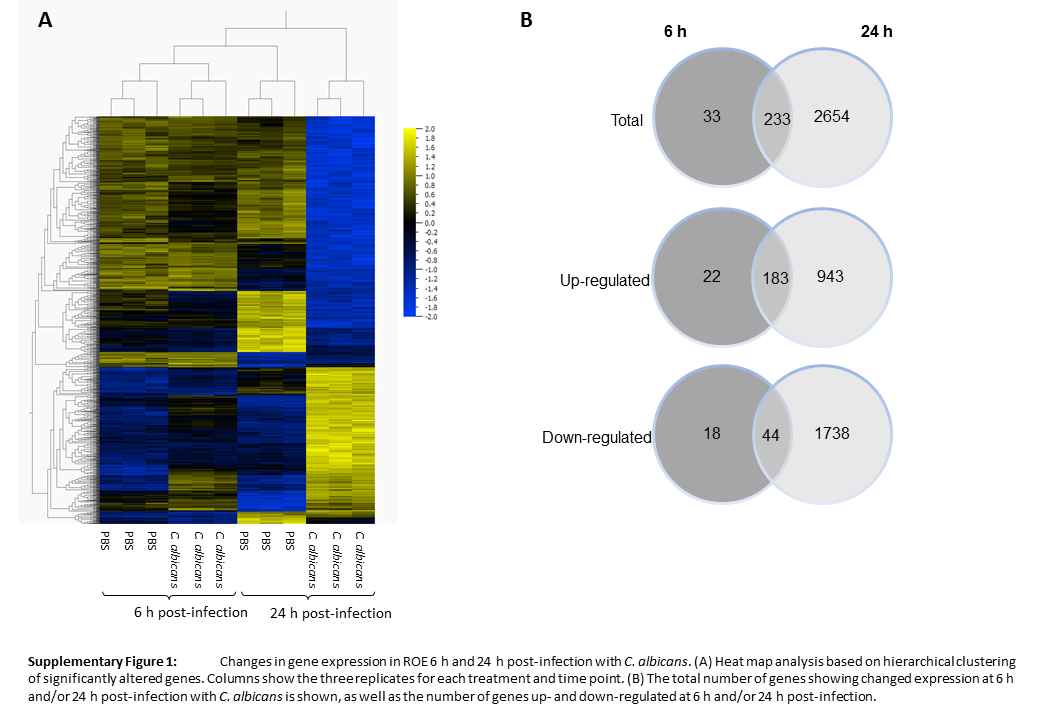

Supplement: Supplementary Data [file supp_jit824_jit824supp_fig1.tif]

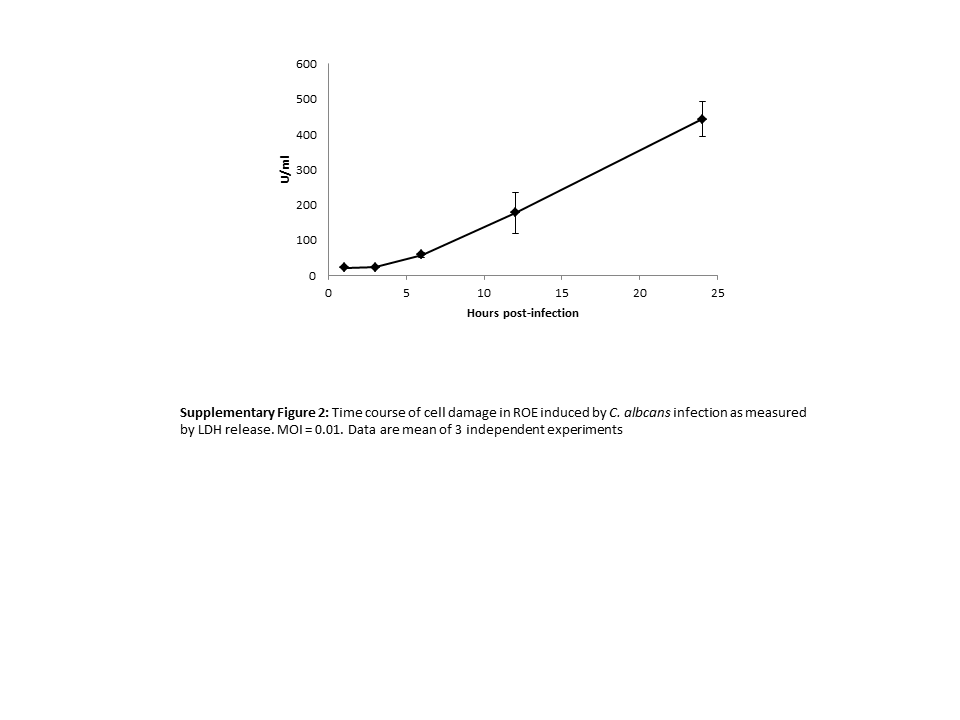

Supplement: Supplementary Data [file supp_jit824_jit824supp_fig2.tif]

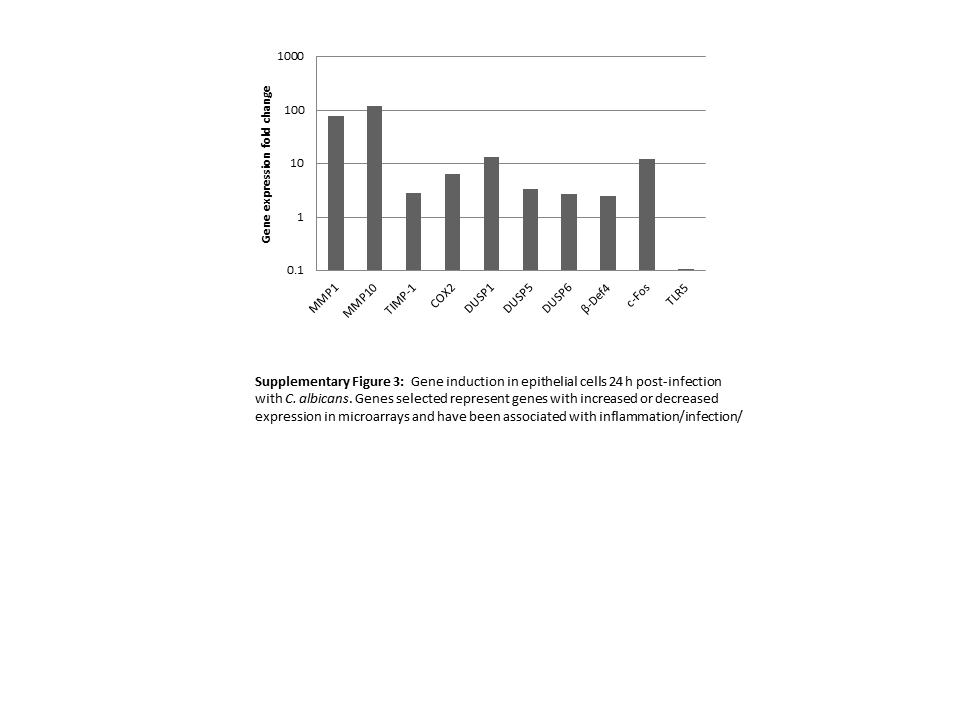

Supplement: Supplementary Data [file supp_jit824_jit824supp_fig3.tif]
